# ICAM-1 ^Kilifi^ variant is not associated with cerebral and severe malaria pathogenesis in Beninese children

**DOI:** 10.1186/s12936-022-04139-0

**Published:** 2022-04-04

**Authors:** Samuel Odarkwei Blankson, Danielle Seri Dadjé, Nadjla Traikia, Maroufou J. Alao, Serge Ayivi, Annick Amoussou, Philippe Deloron, Nicaise Tuikue Ndam, Jacqueline Milet, Leonardo K. Basco, Yaw Aniweh, Rachida Tahar

**Affiliations:** 1 West African Centre for Cell Biology of Infectious Pathogens, Department of Biochemistry, Cell and Molecular Biology, College of Basic and Applied Sciences, University of Ghana, 54 Accra, LG Ghana; 2 Université de Paris, MERIT, IRD, 75006 Paris, France; 3 Pediatric Department, Centre Hospitalier Universitaire Mère-Enfant La Lagune (CHUMEL), Cotonou, Benin; 4 Pediatric Department, Centre National Hospitalo-Universitaire (CNHU), Cotonou, Benin; 5 Pediatric Department, Centre Hospitalo-Universitaire, Suruléré (CHU-Suruléré), Cotonou, Benin; 6 Aix-Marseille Univ, IRD, AP-HM, SSA, VITROME, IHU–Méditerranée Infection, Marseille, France

**Keywords:** *Plasmodium falciparum*, Malaria, cerebral malaria, Polymorphism, ICAM-1, ICAM-1^kilifi^

## Abstract

**Background:**

Cytoadhesion and sequestration of *Plasmodium falciparum* infected red blood cells (iRBC) in the microvasculature of vital organs are a major cause of malaria pathology. Several studies have provided evidence on the implication of the human host intercellular adhesion molecule-1 (ICAM-1) as a major receptor for iRBCs binding to *P. falciparum* erythrocyte membrane protein 1 (PfEMP1) in the development of severe and cerebral malaria. The genetic polymorphism K29M in the immunoglobulin-like domain of ICAM-1, known as ICAM-1^Kilifi^, has been associated with either increased or decreased risk of developing cerebral malaria.

**Methods:**

To provide more conclusive results, the genetic polymorphism of ICAM-1^Kilifi^ was assessed by PCR and sequencing in blood samples from 215 Beninese children who presented with either mild or severe malaria including cerebral malaria.

**Results and conclusions:**

The results showed that in this cohort of Beninese children, the ICAM-1^kilifi^ variant is present at the frequencies of 0.27, similar to the frequency observed in other African countries. This ICAM-1^kilifi^ variant was not associated with disease severity in agreement with other findings from the Gambia, Tanzania, Malawi, Gabon, and Thailand, suggesting no evidence of a direct link between this polymorphism and the pathogenesis of severe and cerebral malaria.

## Background

Malaria presents a heavy burden on people living in endemic areas, with an increase in global mortality to 627,000 in 2020 compared to 405,000 registered in 2019 attributed to the Covid-19 pandemic consequences. Fifteen to 25% of case fatality rate occur among African children with cerebral malaria [1–4]. *Plasmodium falciparum*, the deadliest species, causes several clinical manifestations ranging from asymptomatic and mild infections to life threatening severe malaria, including cerebral malaria. The disease severity has been associated with sequestration of infected red blood cells (iRBCs) within the brain micro-vessels, leading to inflammation, reduction of the blood–brain barrier (BBB) integrity and brain swelling increasing intracranial pressure [3, 5–7]. Furthermore, the accumulation of iRBCs results in microvascular clogging, hypoxia, and activation of inflammatory cytokines, which in turn increase the expression of endothelial cell adherence molecules (eCAM) and accelerate the accumulation of iRBCs capillary beds [8].

Several receptors, including: thrombospondin, cluster of differentiation 36 (CD36), intercellular adhesion molecule 1 (ICAM-1), vascular cell adhesion molecule 1 (VCAM-1), platelet endothelial cell adhesion molecule/cluster of differentiation 31 (PECAM/CD31), neural cell adhesion molecule (NCAM), P and E-selectin, integrin *α*v*β*3, globular C1q receptor (gC1qR), chondroitin sulfate A (CSA), and haemagglutinin (HA), have been shown to be implicated in iRBC cytoadhesion [9–16].

ICAM-1 was found to be up-regulated in endothelial cells and co-localized with iRBCs in brain tissue of children who died from cerebral malaria [17, 18]. *In vitro* static and flow cytoadhesion experiments showed that ICAM-1 mediates attachment of *P. falciparum* iRBCs to cell membrane [19]. Compared to controls, the plasma concentration of soluble form of ICAM-1 is increased in malaria-infected patients [20–23]. ICAM-1 is also expressed constitutively on monocytes, which are often present with the parasites at sites of cerebral micro-haemorrhages in cerebral malaria [24].

ICAM-1 remains a receptor of major interest, and several authors have investigated its role in the pathogenesis of severe malaria [25–31]. Specific ICAM-1 binding to brain microvessels is mediated by the β variant of Duffy-binding like domain (DBLβ) of type A *P. falciparum* erythrocyte membrane protein 1 (PfEMP1) [32]. Recently, parasite strains able to bind both ICAM-1 and Endothelial Protein C Receptor (EPCR) have been isolated and characterized, strengthening the potential role of these two receptors in cerebral malaria [27, 33, 34].

Other studies have focused on the genetic polymorphism of ICAM-1, but these studies have led to contradictory conclusions. A single nucleotide polymorphism (SNP) corresponding to a mutation at locus 56 of the coding sequence, corresponding to position 29 on the mature protein, has been observed at a high frequency in Africa. This non-synonymous coding polymorphism (A/T) leads to a lysine to methionine change (K29M) in the N-terminal domain of ICAM-1. This mutation, known as the ICAM-1^Kilifi^ genotype, was found to predispose children to cerebral malaria in Kenya and Nigeria [35, 36]. However, in other studies conducted in Gambia, Malawi, and Kenya, this mutation was not associated with severe malaria, but was rather associated with protection from severe malaria in a study performed in Gabon [37–40]. Consequently, ICAM-1^Kilifi^ mutation has generated more interest for its potential implications in the mechanisms of pathogenesis of severe and cerebral malaria and has been extensively investigated in cytoadhesion functional studies [41, 42]. Besides, it has also been reported a marginal association of another mutation on exon 6 (rs5498) of ICAM-1with the susceptibility to severe malaria in a case–control study performed in Nigeria [36]. This mutation and the ICAM-1^Kilifi^ have not been found to be associated with susceptibility to severe malaria in whole genome associated study [43]. In the light of these contradictory findings in earlier studies, the frequency of ICAM-1^Kilifi^ was investigated in Beninese children with distinct clinical conditions of malaria, including uncomplicated malaria (UM), severe non cerebral malaria (SNCM) and cerebral malaria (CM), to assess the potential association of ICAM-1^Kilifi^ polymorphism with malaria severity.

## Methods

### Patients

This study was conducted in Cotonou, southern Benin during malaria transmission periods from June to September 2012 and from May to July, 2013. South of Benin is characterized by a subtropical climate, with 2 rainy seasons where *P. falciparum* malaria is endemic with approximately 33 infective bites per person annually [27]. Children less than six years of age presenting at Centre Hopitalier Universitaire Mère-enfant de la Lagune (CHUMEL), Centre National Hospitalier Universitaire Hubert Koutoucou Mega (CNHU-HKM), or to Hôpital Suru-Léré were screened by rapid diagnostic test for malaria (DiaQuick Malaria *P. falciparum* Cassette, Dialab®; Hondastrasse, Austria) and were admitted in the study if they meet the criteria defined by the World Health Organization (WHO) [28]. All malaria cases had microscopically-confirmed *P. falciparum* infection. Three clinical groups were formed including, (1) Cerebral Malaria group (CM) which consisted in children with severe malaria and coma as defined by Blantyre coma score ≤ 2, with the exclusion of any other causes of coma, (2) Severe non-cerebral malaria group (SNCM) which includes children presenting with one or more of the following symptoms; pulmonary edema, acute respiratory distress syndrome, acute kidney failure, abnormal liver function, hemoglobinuria, or severe anemia with absence of coma BCS > 2, (3) Uncomplicated Malaria group (UM): defined as *P. falciparum* parasitaemia with fever, headache, or myalgia without signs of severe malaria and/or evidence of vital organ dysfunction.

After obtaining informed and written consent from children parents or guardians, 2 to 7 ml of venous blood samples were collected into tubes containing citrate phosphate dextrose adenine and 20 µl of each sample were spotted and dried on Whatman (3MM) filter paper. All participants were treated according to the guidelines established by the Beninese Ministry of Health.

### DNA extraction and PCR

DNA was extracted using Chelex® beads [44]. Briefly, a 2 mm diameter disc was cut from Whatman filter paper and incubated at 4 °C overnight in 0.5 mL of phosphate-buffered saline (PBS) containing 0.5% saponin. The filter paper was washed twice in saponin-free PBS, placed in 100 µl of distilled water containing 10% Chelex® (Biorad, Marnes-la-Coquette, France), then incubated at 100 °C for 20 min to elute DNA. Tubes were centrifuged at 12000x*g*, and the supernatant transferred to a new tube. One microlitre of this suspension was used to perform PCR for immunoglobulin (Ig)-like domain of *icam-1* gene using the primers and PCR conditions described by Fernandez-Reyes et al. [35]. The 263-bp amplified fragment spanning codon 29 was sequenced from the 5′- and 3′-ends using an automated DNA sequencer (ABI Prism; Perkin Elmer Corp, Eurofins, Paris, France).

### Statistical analysis

The statistical analysis was performed on Prism v7 software (GraphPad Software, Inc., San Diego, CA, USA). Quantitative variables were compared between the three groups using the non-parametric Kruskall-Wallis test. Association between the ICAM-1^Kilifi^ genotype and clinical groups was performed using a chi-square test to compare genotypes as well as allele frequencies. P-value from the global chi-square assessing if there is at least one difference between the three groups are reported. The level of statistical significance was set at 0.05.

## Results

### Clinical and biological characteristics of patients

The base line characteristics, the clinical and the biological parameters of the children enrolled in the study are summarized in (Table 1). Briefly, we included 74 children with cerebral malaria (CM), 71 with severe non cerebral malaria (SNCM) and 70 with uncomplicated malaria (UM). There was no significant difference in age, male to female ratio, temperature, and parasitaemia. However, haemoglobin level was significantly different between clinical groups with a P-value of P < 0.0001. As expected, the deaths occurred among children in the group of CM and SNCM with a high mortality rate of 43% in children with CM compared to 17% in children with SNCM, P = 0.014.

**Table 1:** Clinical and biological characteristics of *P. falciparum-*infected children enrolled in the study

Characteristics	Clinical groups		
	Cerebral malaria	Severe non-cerebral malaria	Uncomplicated malaria
Number of patients	74	71	70
Age, median (range), months	36 (5–72)	30 (9–60)	36 (5–60)
Sex ratio (male/female)	1.46 (44/30)	0.86 (33/38)	1.12 (37/33)
Geometric mean parasitaemia (95% CI; [range]) asexual parasites/µl	44,400 (15,100–34,900) [2,720–864,000]	21,800 (32,700–83,900) [156–650,000]	22,300 (16,000–42,900) [75–247,000]
Body temperature, median (range) °C	38.5 (35.5–40.9)	38.0 (36.0–41.0)	38.5 (36.0–40.7)
Haemoglobin, median (range) g/dl*	6.0 (0.8–12.9)	8.6 (3.1–11.5)	8.6 (5.0–13.6)
Blantyre score, median (range)	2 (0–2)	3.5 (3–5)	5 (–)
Number of deaths (%)*	32 (43.2)	12 (16.9)	0

^*^The difference between different groups was statistically significant for haemoglobin (*P* < 0.0001) and the number of deaths (*P* = 0.014). 95% CI, 95% confidence interval

### Allelic frequency at ICAM- 29 position of enrolled children

Fragments of the N-terminal immunoglobulin-like domain of ICAM-1, were successfully amplified and sequenced in 215 individuals after genomic DNA extraction from blood samples. Hardy Weinberg Equilibrium test performed in UM sample to detect potential population stratification or problems in genotyping showed no deviance from the expected frequencies of genotypes (P = 0.78). The allelic frequencies of the mutant were 0.22 in CM group and 0.3 in both SNCM and UM groups. Even if we observe a higher frequency of wild type K29/K29 genotype in cerebral malaria group, the comparison of allelic and genotypic frequencies between the clinical groups showed no significant difference (respectively P = 0.18 And P = 0.19 global chi2 test) in the proportions of wild-type and mutant alleles or genotypes. The allelic and genotypic frequencies are presented in (Table 2).

**Table 2:** Genotypes and allelic frequency at ICAM-1- 29 loci

Clinical group	Genotype (n, %)			Frequency	
	K29/K29	K29/M29	M29/M29	K29	M29
Cerebral malaria (n = 74)	49 (66.2)	17 (23.0)	8 (10.8)	0.78	0.22
Severe non-cerebral malaria (n = 71)	40 (56.3)	18 (25.4)	13 (18.3)	0.69	0.31
Uncomplicated malaria (n = 70)	36 (51.4)	26 (37.1)	8 (11.4)	0.70	0.30
Total (n = 215)	125 (58.1)	61 (28.4)	29 (11.4)	0.72	0.27

K29/K29 are wild-type homozygous patients; K29/M29 are heterozygous individuals; M29/M29 indicate homozygous patients with the ICAM-1^Kilifi^ mutation. n, number of patients or samples

## Discussion

The biological mechanisms driving towards severe malaria pathology involve parasite virulence, host immunological backgrounds and host genetic factors. Among these factors parasite proteins expressed on the surface of erythrocytes such as PfEMP1 and host endothelial receptors play a major role. The results of the present study which aimed to investigate the possible association between the ICAM-1^Kilifi^ genotype and the predisposition to cerebral or severe malaria in African children from Benin show a high frequency of this mutation which reached (0.27%) consistent with earlier finding in other African countries. However, no association between the ICAM-1^Kilifi^ variant and the occurrence of cerebral or severe malaria has been found. These results are in agreement with those found in studies obtained on 2685 Gambian children, 200 Gabonese and 477 Thai individuals, but in contradiction to those of the initial study carried out on 547 Kenyan children [35, 37–39]. The importance of ICAM-1 receptor in the pathogenicity of severe malaria and the unequal distribution of the ICAM-1^Kilifi^ which reached frequencies between 20 to 30% in high malaria transmission region of Africa, around 5% in lower transmission area of East-Asia, and at only 0.4 to 1.1% in non-endemic area suggest a selective pressure exerted by malaria at this locus [38].

Functional studies showed that the iRBCs binding site is part of the two first domains of ICAM-1, overlaps but is distinct from that of Lymphocyte functional associated Antigens (LFA), Macrophage receptor 1 (Mac-1) and human rhinovirus [45, 46]. Indeed, another study showed that the conformational changes produced by the ‘Kilifi polymorphism’ occur at the L43 loop of domain 1 ICAM-1 and the monoclonal antibody 15.2 that maps to this region blocks the binding of iRBCs to both ICAM-1^ref^ and ICAM-1^Kilifi^ forms. Furthermore, both static and dynamic cytoadhesion experiments showed that phenotypic differences in the binding characteristics between these two ICAM-1 variants may depend on *P. falciparum* strains used in experimental assays. Thus, *P. falciparum* ITG iRBCs binds equally to ICAM-1^Ref^ and ICAM-1^Kilifi^, however *P. falciparum* A4 iRBCs strain binds weakly to ICAM-1^Kilifi^ [41]. The difference in binding was more important in the dynamic assays, suggesting that ICAM-1^Kilifi^ may select high-affinity binding parasites at sequestration sites within the brain microvessels [47]. These observations were confirmed later using three different parasite lines (ItG, JDP8, A4) with different binding ability to wild type ICAM-1^ref^ to evaluate their adherence-capacities to a panel of mutant ICAM-1 proteins (ICAM-1^K29M(Kilifi)^, ICAM-1^S22/A^, ICAM-1^L42/A^ and ICAM-1^L44/A^) both under flow and static conditions. The results showed that iRBCs binding to some ICAM-1 mutants was reduced to 80% or completely abolished for some isolates, while the iRBCs binding to ICAM-1^Kilifi^ was reduced in only 50% of isolates, emphasizing the importance of parasite PfEMP1 variants used in the interaction with ICAM-1 [48].

More recently, the ICAM-1^Kilifi^ mutation was shown to be significantly associated with child hospitalisation in Tanzania supporting the link between this mutation with malaria severity, but independent from the cytoadherence pattern of iRBCs on ICAM-1, which can also depend on binding of these isolates to other receptors rather than ICAM-1 in these [49].

These findings may explain the contradictory results on the association of this variant with cerebral malaria [37, 39, 40] which, seems to also depend on PfEMP-1 and non PfEMP1 variants expressed at the surface of iRBCs and support the frequency-dependent model of selection explanation proposed by Fry et al. [38]. a mechanism which have been proposed among others to underlie host-parasite evolutionary dynamic. This model is based on an established equilibrium between polymorphism frequency in human and that of parasite strains due to competition between strains preferring binding on either ICAM-1^Kilifi^ or ICAM-1^ref^, the change in host in allele frequency will favor the expansion of the corresponding high binding parasite strains which will select in return against the most frequent allele in host bringing the system to equilibrium at which all individuals will have the same risk of developing severe or cerebral malaria irrespective of their ICAM-1 genotype [38]. The ICAM-1^Kilifi^ mutation was also linked to the protection from highly prevalent non-malarial febrile illness in sub-Saharan Africa such as sepsis, suggesting that this polymorphism play a role in the modulation of inflammatory response to pathogens by ICAM-1 and may subsequently explain the high frequency of this polymorphism within African populations [49, 50].

## Conclusion

The results of the present study indicate that ICAM-1^Kilifi^ polymorphism is not directly associated to severe or cerebral malaria development. However, a role in the pathogenesis, depending on the parasite variants implicated in the interaction with ICAM-1 ^Kilifi^ cannot be completely excluded.

## Data Availability

Sequencing data generated and analysed in this study are available in this manuscript. Other data can be obtained from the corresponding author on reasonable request.

## Abbreviations

CM: Cerebral malaria
EPCR: Endothelial Protein C Receptor
ICAM-1: Intercellular adhesion molecule-1
iRBCs: Infected red blood cells
PfEMP1: *Plasmodium* *falciparum* Erythrocyte membrane protein 1
SNP: Single nucleotide polymorphism
SNCM: Severe non cerebral malaria
UM: Uncomplicated malaria
